# Appropriateness of the Prescription and Use of Medicines: An Old Concept but More Relevant than Ever

**DOI:** 10.3390/ijerph20032700

**Published:** 2023-02-02

**Authors:** Manuela Casula, Ilaria Ardoino, Carlotta Franchi

**Affiliations:** 1 Epidemiology and Preventive Pharmacology Service (SEFAP), Department of Pharmacological and Biomolecular Sciences, University of Milan, 20133 Milan, Italy; 2 IRCCS MultiMedica, Sesto S. Giovanni, 20099 Milan, Italy; 3 Laboratory of Pharmacoepidemiology and Human Nutrition, Department of Health Policy, Istituto di Ricerche Farmacologiche Mario Negri IRCCS, 20156 Milan, Italy; 4 Italian Institute for Planetary Health (IIPH), 20156 Milan, Italy

The availability of drugs to treat diseases, control symptoms, or prevent their onset is one of the most important resources for maintaining health. Over time, the need to navigate through the ever-increasing therapeutic armamentarium and the need to optimize available resources has highlighted the importance of using these tools correctly.

In the 1990s, the term ‘appropriateness’ related to health care began to emerge [1,2]. Since then, many studies have been conducted to evaluate specific cases of inappropriateness and to estimate the consequences on patients’ health; moreover, many interventions have been implemented to achieve improvement [3,4]. However, the evidence shows us that there is still a long way to go.

Just to give a few examples, the prescription of antibiotics continues to show high rates of inappropriateness [5,6]. Antibiotics are among the most widely used pharmacological agents, but have been frequently shown to be over prescribed and used inappropriately [7,8]; this contributes to the problem of antibiotic resistance, which increases morbidity and mortality and causes substantial economic burden [9,10]. Despite increasing efforts to reduce the inappropriate prescription of antibiotics, political strategies, and public engagement [11], the problem still appears to be growing. In 2019, WHO listed antimicrobial resistance as one of the top ten threats to global health [12]. A systematic evaluation of antibiotic consumption across 76 countries from 2000 to 2015 reported that overall use per capita increased by 26.2% in first-line or second-line antibiotics and by 90.9% in antibiotics with restricted indications (due to higher resistance potentials) [13]. A meta-analysis conducted on a total of 123 point-prevalence estimates from 37 countries between 1985 and 2019 [14] showed that the percentage of residents in long-term aged care facilities receiving an antibiotic on a single day ranged from 0.7% to 17.3%. The percentage of appropriate courses ranged between 9.5% to 60.3% and decreased every year. Of note, this picture is strongly related to the increased antibiotic resistance-associated morbidity and mortality. A very recent study estimated 4.95 million deaths associated with drug-resistant infections in 2019. Compared with all underlying causes of death, antimicrobial resistance was the third leading cause of death in 2019, just after ischemic heart disease and stroke [15].

Another class of drugs that is widely used and often singled out for being overprescribed and used inappropriately are proton pump inhibitors (PPIs) [16,17]. The introduction of PPIs into clinical practice thirty years ago has greatly improved the therapeutic approach to acid-related diseases for their well-recognized efficacy and safety. However, despite well-defined indications, the use of PPIs continues to grow every year in both Western and Eastern countries, raising serious queries about appropriate prescription. The market for these drugs has progressively increased to the point that it is estimated that over 110 million PPI prescriptions are filled each year in the US [18]. It has been calculated that up to 50% of PPIs are prescribed inappropriately in general medical wards and in general practitioners’ practice, as patients failed to meet proper indications for PPI therapy [17,19,20,21]. Indeed, the most common reasons for inappropriate prescription are ulcer prophylaxis in low-risk patients or in patients taking steroids alone or anti-coagulants without any risk factor, or the treatment of functional dyspepsia continuing indefinitely without any periodic re-evaluation [16]. Frequently, PPIs are perceived by doctors themselves as a harmless and relatively inexpensive remedy for any digestive trouble. However, despite a relatively good safety profile, the use of PPIs is not without risk, especially if continued over the long term. Indeed, prolonged treatment with PPIs has been associated with increased risk of infections, bone fractures, and renal damage, malabsorption of vitamins and minerals, and other complications [22]. Moreover, in those patients already treated with PPIs before a hospitalization, often hospital doctors continue to prescribe these drugs at discharge without any critical evaluation, so that general practitioners think that the prescription is appropriate and must be pursued in the long term [23].

The last example we would like to mention is the use of benzodiazepines (BZD) in elderly people. The avoidance of long half-life BZD (e.g., diazepam, chlordiazepoxide) is recommended because of concerns such as excessive sedation and an increased risk of falls and fractures [24]. Despite evidence of many potential risks, the use of BZD among older adults to manage insomnia, delirium, and dementia is common. National estimates suggest that, in 2015, 8.7% of Americans older than 65 were prescribed BZD within the past year [25], and some studies reported even higher prevalence [26,27], although less than half of BZD prescriptions in this age group are considered appropriate [24,28]. In an Italian study conducted in more than 100 internal medicine and geriatric wards, the authors highlighted that, among 4681 patients discharged, 15% were discharged with BDZs, and 62% of them were inappropriately prescribed, being prescribed with BDZ when it should be avoided (45%), at higher doses than recommended (31%), or with no appropriate clinical conditions (19%) [28]. Some authors suggested that the reasons why inappropriate psychotropic medications continue to be prescribed include the fact that healthcare professionals may be unaware of which psychotropic agents are inappropriate and why. Additionally, the lack of a complete consensus on inappropriate psychotropic medications can have a role, highlighting the need for further studies to explore the real-life practice, quantify the clinical risks, and identify possible risk factors [29].

This third example, in particular, allows us to emphasize two aspects. Firstly, in terms of therapeutic appropriateness, the elderly is a particularly high-risk group. The presence of several concomitant diseases requires the use of numerous drugs (a condition referred to as polypharmacy) [30,31,32], and this increases the possibility of inappropriateness due to drug–drug or drug–disease interactions. In addition, age-associated physiological modifications, changes in pharmacokinetics and pharmacodynamics, and frailty increase the risk of developing adverse effects [33]. In this population, adherence to therapy is also a crucial aspect [34]. The high number of medications, together with compromising conditions such as dementia, memory loss and confusion, may prevent the patient from following the prescribed therapy appropriately and thus deriving the maximum benefit [35,36]. Secondly, we cannot neglect to consider the potential detrimental impact of the recent COVID-19 pandemic. Real-world evidence suggests that medication-related problems have been exacerbated [37,38]. The burden of the health emergency fell heavily on general practitioners and other health professionals, who necessarily had to reorganize their health care activities, often devoting less time to the routine management of the treatment of diseases other than SARS-CoV-2 [39]. The pandemic has also increased the use and misuse of some medications, such as BZD [40,41], and increased self-medication behaviors [42].

Those mentioned are just a few cases, but the literature is full of examples of how inappropriate pharmacological treatment is still a widespread problem that impacts on patients’ health and leads to unnecessary costs for healthcare systems [43,44,45,46]. Moreover, reducing inappropriate drug use should be seen as a strategy to curb the environmental impact of medications, thus containing the devastating effects of climate change on human and planetary health. Within this framework, prescriptive appropriateness is an ongoing challenge, based on evolving knowledge, influenced by old and well-known factors as well as new circumstances. Therefore, the ultimate goal of achieving patient well-being with the efficient use of available resources cannot be pursued without the continuous collection of data, taking advantage of real-life research approaches in particular. The accuracy and timeliness of this information is a valuable element for decision-makers in order to promote the best strategies, make the appropriate tools available, and support the clinician, who is responsible for the individual therapeutic decision—in the context of a comprehensive patient assessment. As stated by Stephen A. Buetow, ‘to prescribe appropriately therefore is a science and an art; the challenge is to get the balance right’ [1].

## Data Availability

Not applicable.
